# Tumor suppressor role of miR-3622b-5p in ERBB2-positive cancer

**DOI:** 10.18632/oncotarget.14968

**Published:** 2017-02-01

**Authors:** Mingjie Lu, Tongshan Wang, Mingfeng He, Wenfang Cheng, Ting Yan, Zebo Huang, Lan Zhang, Huo Zhang, Wei Zhu, Yichao Zhu, Ping Liu

**Affiliations:** ^1^ Department of Oncology, First Affiliated Hospital of Nanjing Medical University, Nanjing 210029, PR China; ^2^ Department of Anesthesiology, First Affiliated Hospital of Nanjing Medical University, Nanjing 210029, PR China; ^3^ Department of Gastroenterology, First Affiliated Hospital of Nanjing Medical University, Nanjing 210029, PR China; ^4^ Safety Assessment and Research Center for Drug, Pesticide and Veterinary Drug of Jiangsu Province, Nanjing Medical University, Nanjing 211166, PR China; ^5^ Department of Physiology, Nanjing Medical University, Nanjing 211166, PR China; ^6^ State Key Laboratory of Reproductive Medicine, Nanjing Medical University, Nanjing 211166, PR China

**Keywords:** miR-3622b-5p, ERBB2, cancer, proliferation, apoptosis

## Abstract

Over-expression or amplification of ERBB2 is observed in multifarious carcinomas. However, the molecular mechanism of ERBB2 downregulation in ERBB2-positive cancers remains obscure. This experiment investigated the suppressive role of miR-3622b-5p in ERBB2-positive breast and gastric cancers. The luciferase activity of ERBB2 3′-untranslated region-based reporters constructed in HEK-293T, SK-BR-3 and MCF-10A cells suggested that ERBB2 was the target gene of miR-3622b-5p. Over-expressed miR-3622b-5p reduced the protein level of ERBB2, weakened the activation of mTORC1/S6, and induced the apoptosis of ERBB2-positive cancer cells. MiR-3622b-5p was significantly down-regulated in breast and gastric cancer tissues. This down-regulation in ERBB2-positive breast and gastric cancer tissues was more obvious than that in ERBB2-negative breast and gastric cancer tissues. MiR-3622b-5p turned ERBB2-positive cancer cells more vulnerable to the apoptosis induced by cisplatin and 5-fluorouracil. Taken together, miR-3622b-5p is involved in the proliferation and apoptosis of human ERBB2-positive cancer cells via targeting ERBB2/mTORC1 signaling pathway.

## INTRODUCTION

Over-expression of human epidermal growth factor receptor 2 (ERBB2 or Her2) contributes to the malignant progression of tumors [1], especially the tumors of colon, bladder, ovary, endometrium, lung, uterine cervix, head and neck, and stomach [2–8]. As a molecular abnormality triggered by gene amplification, ERBB2 over-expression occurs in about 25 % of breast carcinomas and a subset of aggressive tumors [9]. Mounting evidence proves that ERBB2 over-expression in gastric cancer leads to poor prognosis [10]. Although ERBB2-targetd therapy (trastuzumab or herceptin) has been used to treat ERBB2-overexpressing tumors [11, 12], patients’ drug resistance thwarts its clinical wide spread [13].

MicroRNAs (miRNAs) are essential to cancer cell survival, proliferation, differentiation, migration, invasion and metastasis [14–16]. MiRNA subtypes, including miR-548d-3p, miR-559, miR-125a, miR-125b, miR-205, miR-155 and miR-4728, target ERBB2 and are downregulated in cancers [17–20]. However, the specific mechanism of miRNA in ERBB2-positive cancers is still unknown. This experiment demonstrated for the first time that the level of miR-3622b-5p was inversely correlated with the level of ERBB2 expression in human ERBB2-positive tumors, and that miR-3622b-5p induced the apoptosis of breast and gastric cancer cells by repressing ERBB2 expression. MiR-3622b-5p made ERBB2-positive cancer cells more susceptible to the apoptosis induced by cisplatin and 5-fluorouracil. Collectively, these findings conclude that miR-3622b-5p is a regulator of ERBB2 in ERBB2-positive cancers.

## RESULTS

### ERBB2 as the target gene of miR-3622b-5p

**Figure 1:**
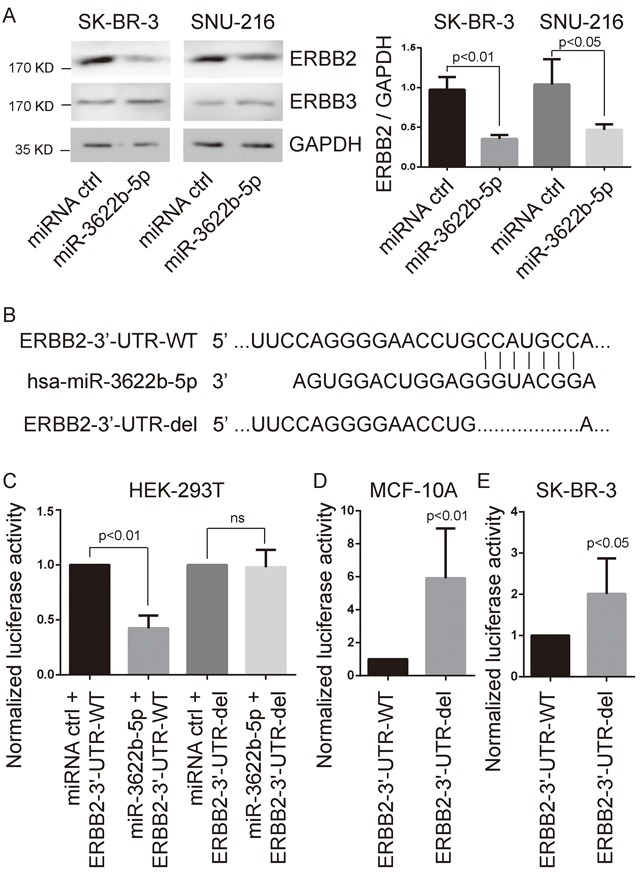
ERBB2 as target of miR-3622-5p **A**. Western blot analysis showing suppressed ERBB2 protein levels in SK-BR-3 breast cancer cells and SNU-216 gastric cancer cells after miR-3622-5p over-expression. The over-expression of miR-3622-5p does not alter the expression level of ERBB3. GAPDH as the internal control. Graphical presentation in the right panel. **B**. The seed sequence of miR-3622b-5p is complementary to the 3′-UTR of ERBB2. **C**. Luciferase assay showing reduction in reporter activity (relative luciferase units) after co-transfection of wild type ERBB2 3′-UTR (ERBB2-3′UTR-WT) or the fragment of ERBB2 3′-UTR lacking the candidate miR-3622b-5p binding sequence (ERBB2-3′-UTR-del) with miR-3622-5p into HEK-293T cells. ns, no significance. **D**. Luciferase assay showing reduction in reporter activity after transfection of ERBB2-3′UTR-WT or ERBB2-3′-UTR-del in MCF-10A cells. **E**. Luciferase assay showing reduction in reporter activity after transfection of ERBB2-3′UTR-WT or ERBB2-3′-UTR-del in SK-BR-3 cells.

TargetScan Human (http://www.targetscan.org) suspects that ERBB2 is the target gene of the miR-18-5p, miR-125-5p, miR-133a-3p or miR-3622b-5p. To explore whether ERBB2 is the target gene of those miRNAs, we transfected miR-18-5p, miR-125-5p, miR-133a-3p or miR-3622b-5p mimic and miRNA mimic control into SK-BR-3 or SNU-216 cells. Consequently, the expression of ERBB2 in miR-3622b-5p-transfected cells, not in cells transfected with miR-18-5p, miR-125-5p or miR-133a-3p, was more significantly depressed than that in control cells (Figure 1A and Supplementary Figure 1). Meanwhile, the over-expression of miR-3622b-5p did not alter the expression level of ERBB3 in SK-BR-3 and SNU-216 cells (Figure 1A).

Next, we constructed the luciferase reporter vectors at the putative ERBB2 3′-UTR target site (ERBB2-3′-UTR-WT) or the fragment of ERBB2 3′-UTR lacking the candidate miR-3622b-5p binding sequence (ERBB2-3′-UTR-del) for the miR-3622b-5p downstream of the luciferase gene (Figure 1B). Luciferase reporter vectors, miR-3622b-5p mimic or the miRNA mimic control were transfected into HEK-293T cells, respectively. For HEK-293T cells, a significant decrease of luciferase activity became notable when ERBB2-3′-UTR-WT was co-transfected with miR-3622b-5p mimic, but this decrease did not show up in the ERBB2-3′-UTR-del group (Figure 1C). After transfecting luciferase reporters into MCF-10A or SK-BR-3 cells, ERBB2-3′-UTR-del up-regulated the relative luciferase activity more obviously compared to the ERBB2-3′-UTR-WT group (Figure 1D and 1E). Taken together, these results show that ERBB2 is the target gene of miR-3622b-5p.

### MiR-3622b-5p suppressed mTOR signaling in ERBB2-positive cancer cells

**Figure 2:**
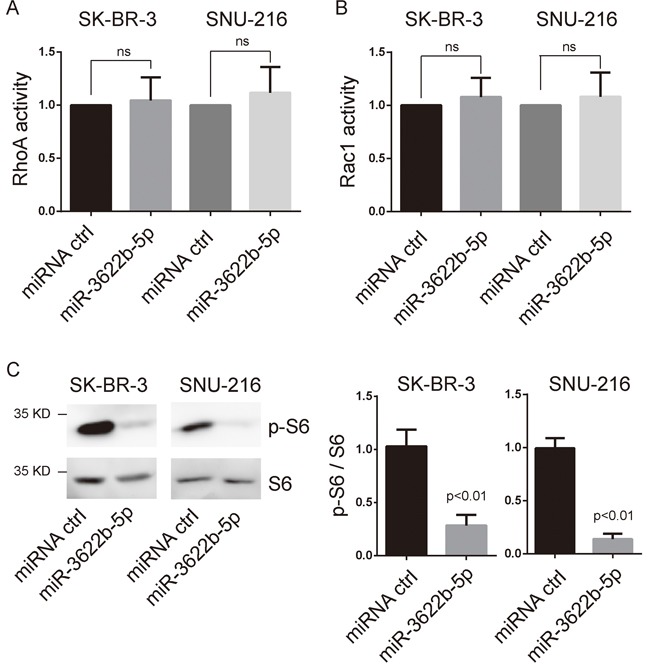
MiR-3622b-5p suppresses mTORC1 signaling **A**. and **B**. miR-3622b-5p overexpression did not alter the activation of RhoA and Rac1 in SK-BR-3 breast cancer cells and SNU-216 gastric cancer cells. The RhoA or Rac1 relative active levels were normalized to the average value of SK-BR-3 or SNU-216 cells transfected with control miRNA. ns, no significance. **C**. miR-3622b-5p overexpression suppresses the phosphorylation of S6 in SK-BR-3 breast cancer cells and SNU-216 gastric cancer cells. Graphical presentation in the right panel.

ERBB2 induction of migration and invasiveness of cancer cells require the activation of small GTPases and mTORC1 signaling [21–23]. We examined the activation of RhoA, Rac1 and mTORC1 signaling in miR-3622b-5p-transfected cells and control ERBB2-positive cells. As a result, the activations of RhoA and Rac1 were not significantly changed in miR-3622b-5p-transfected cells compared to those in control SK-BR-3 or SNU-216 cells (Figure 2A and 2B). However, phosphorylation of S6, the direct downstream of mTORC1 signaling, was obviously down-regulated in miR-3622b-5p-transfected cells (Figure 2C). These results suggest that miR-3622b-5p down-regulates the mTORC1 signaling, not Rho signaling, in ERBB2-positive cancer cell lines.

### MiR-3622b-5p inhibited the proliferation and induced the apoptosis of ERBB2-positive cancer cells

**Figure 3:**
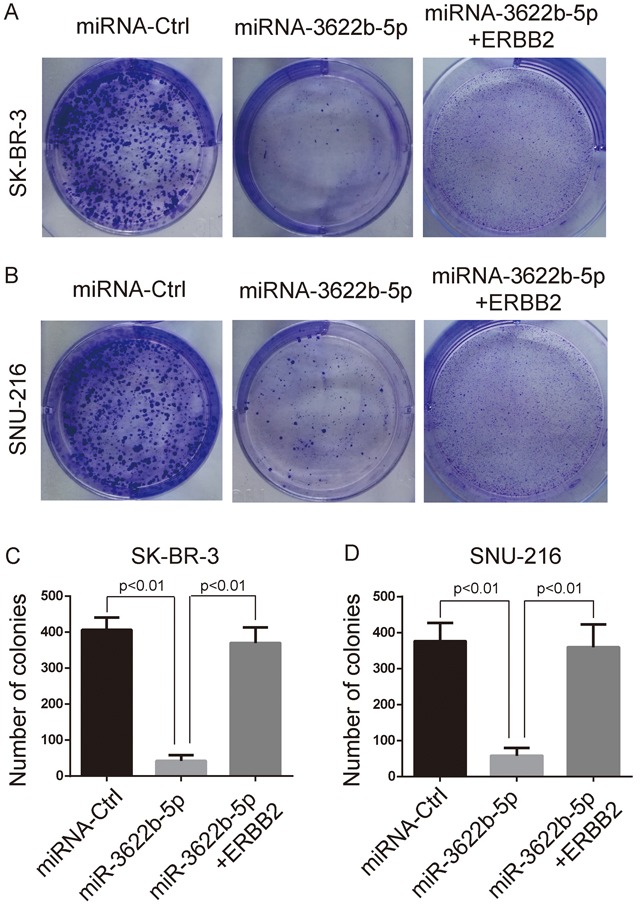
MiR-3622b-5p suppresses the colony formation ability of ERBB2-positive cancer cells MiR-3622b-5p over-expression reduced the colony formation ability of SK-BR-3 breast cancer cells **A**. and **C**. or SNU-216 gastric cancer cells **B**. and **D**. which could be reversed by the over-expression of ERBB2 lacking the 3`-UTR. The number of colonies was counted and shown in panel C and D.

**Figure 4:**
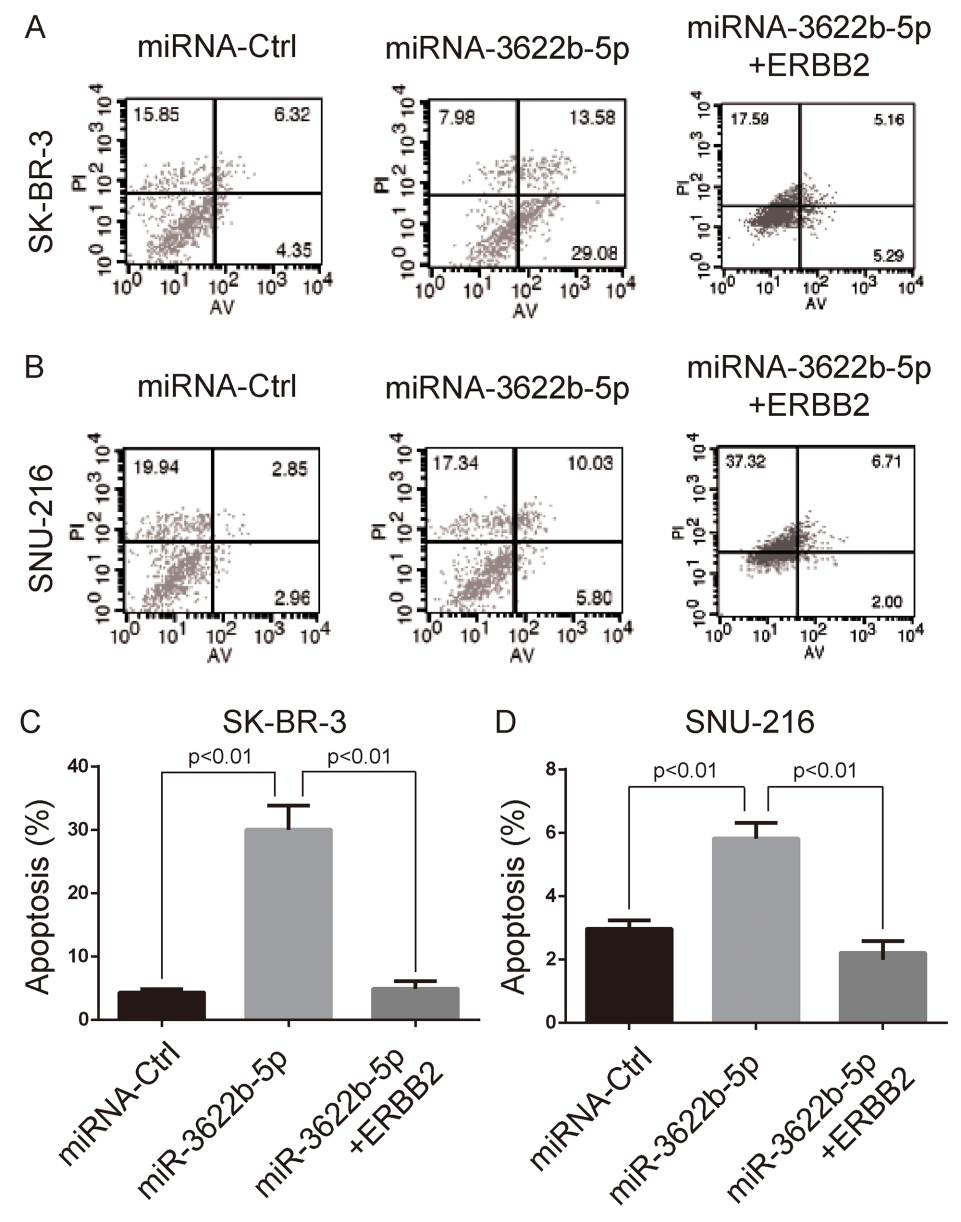
MiR-3622b-5p induces the apoptosis of ERBB2-positive cancer cells MiR-3622b-5p over-expression induced the apoptosis of SK-BR-3 breast cancer cells **A**. and **C**. or SNU-216 gastric cancer cells **B**. and **D**. which could be reversed by the over-expression of ERBB2 lacking the 3`-UTR. The percent of apoptotic cells (AnnexinV-positive and PI-negative cells) was shown in panel C and D.

MTORC1 controls the cell growth and body homeostasis [24]. Clonogenic assay revealed that SK-BR-3 and SNU-216 cells transfected with miR-3622b-5p mimic exhibited a significant decrease of clones, and this decrease could be reversed by the over-expression of ERBB2 lacking the 3`-UTR (Figure 3). Moreover, SK-BR-3 and SNU-216 cells transfected with miR-3622b-5p mimic exhibited significant apoptosis that could be reversed by the over-expression of ERBB2 lacking the 3`-UTR (Figure 4). Collectively, these results suggest that miR-3622b-5p inhibits the proliferation and induces the apoptosis of ERBB2-positive cancer cells.

### MiR-3622b-5p was down-regulated in ERBB2-positive cancer tissues

**Figure 5:**
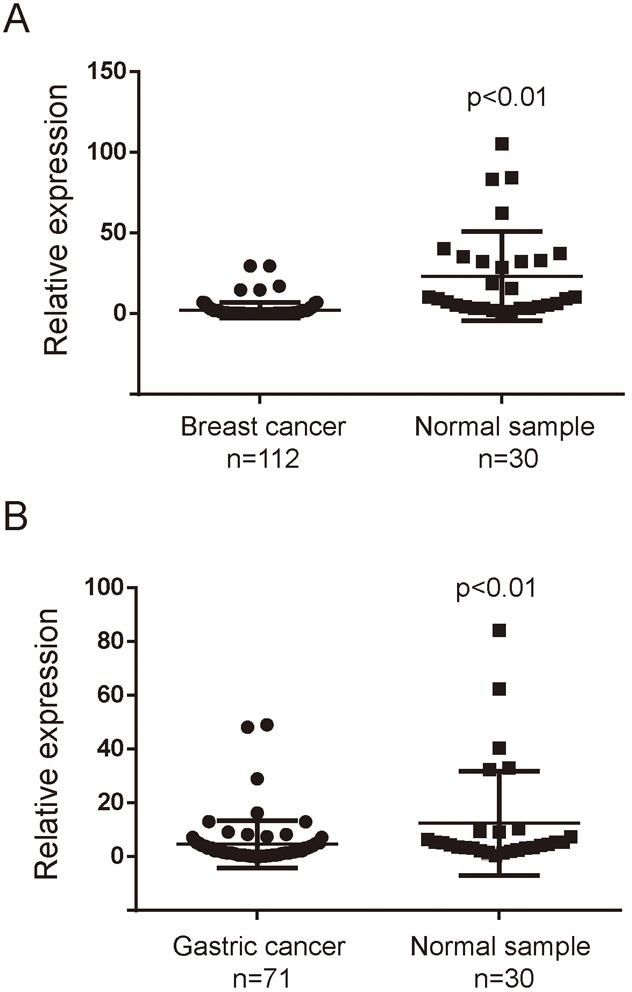
MiR-3622b-5p is down-regulated in cancer tissues The miR-3622-5p expression was suppressed in a majority of breast cancer samples when compared to normal breast samples **A**. or in a majority of gastric cancer samples when compared to normal gastric samples **B**. The miRNA relative expression levels were normalized to the average value of breast cancer samples (A) or gastric cancer samples (B).

MiR-3622b-5p expression in breast cancer tissues and gastric cancer tissues was examined by quantitative real-time PCR. To analyze the expression in breast cancer tissues, samples were made with cancer tissues of 112 patients and adjacent normal tissues of 30 patients. To analyze the expression in gastric cancer tissues, samples were made with gastric tissues of 71 patients and tumor adjacent normal tissues of 30 patients. According to the results, miR-3622b-5p expression was significantly down-regulated in cancer tissues, compared with that in tumors’ adjacent normal tissues, respectively (Figure 5A and 5B). We also found that miR-3622b-5p expression was significantly down-regulated in SK-BR-3 breast cancer cells compared with that in MCF-10A normal human mammary epithelial cells (Supplementary Figure 2).

**Figure 6:**
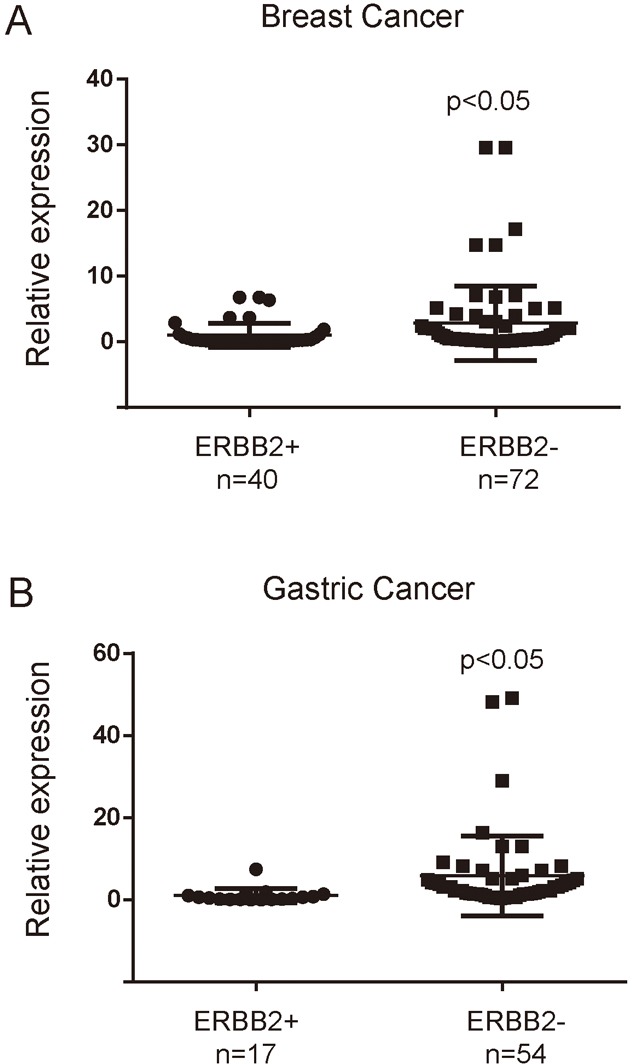
MiR-3622b-5p is down-regulated in ERBB2-positive cancer tissues The miR-3622b-5p expression was suppressed in a majority of ERBB2-positive breast cancer samples when compared to ERBB2-negative breast cancer samples **A**. or in a majority of ERBB2-positive gastric cancer samples when compared to ERBB2-negative gastric cancer samples **B**. The miRNA relative expression levels were normalized to the average value of ERBB2-positive breast cancer samples (A) or ERBB2-positive gastric cancer samples (B).

Meanwhile, the association between miR-3622b-5p and ERBB2 expression was explored by testing the miR-3622b-5p expression in 40 patients’ ERBB2-positive breast cancer tissues and 72 patients’ ERBB2-negative breast cancer tissues, and in 17 patients’ ERBB2-positive gastric cancer tissues and 54 patients’ ERBB2-negative gastric cancer tissues. The results showed that miR-3622b-5p was significantly down-regulated in human ERBB2-positive cancer tissues, compared with that in ERBB2-negative cancer tissues (Figure 6A and 6B). Taken together, these findings indicate that the miR-3622b-5p level is inversely correlated with ERBB2 expression in epithelial tumors.

### MiR-3622b-5p turned ERBB2-positive cancer cells more vulnerable to the apoptosis induced by cisplatin and 5-fluorouracil

**Figure 7:**
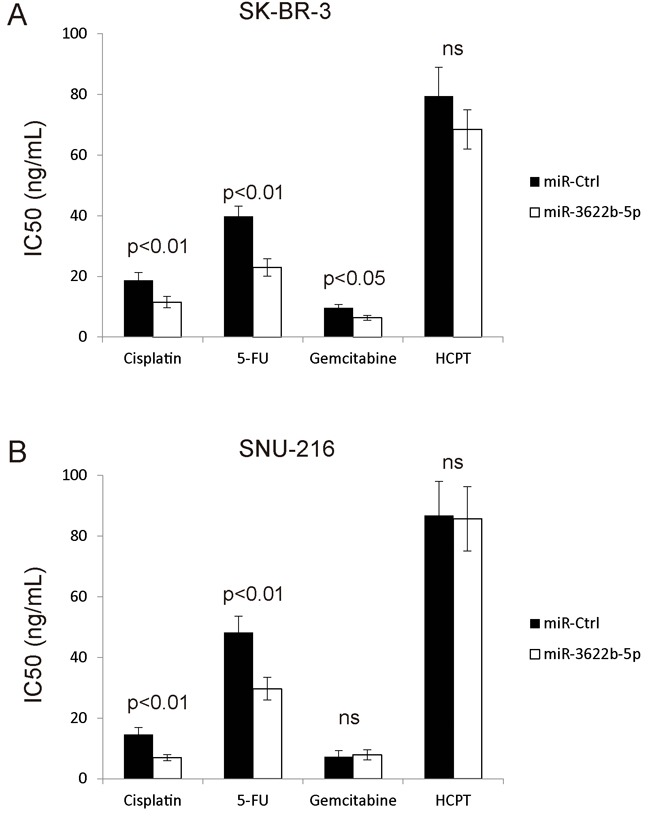
MiR-3622b-5p sensitizes cells to chemotherapy drugs Over-expression of miR-3622b-5p in SK-BR-3 breast cancer cells **A**. or SNU-216 gastric cancer cells **B**. significantly increased cells’ sensitivity to cisplatin and 5-fluorouracil (5-FU) compared to control miRNA. Cells were treated with chemotherapeutic drugs 24 h after miR-3622b-5p transfection and the cell viability was assessed 48 h after chemotherapeutic drugs treatment. 5-FU, 5-fluorouracil. HCPT, hydroxy camptothecin. ns, no significance.

Since miR-3622b-5p induces the apoptosis of ERBB2-positive cancer cells, it is proposed that miR-3622b-5p might evoke the apoptosis of ERBB2-positive cancer cells by weakening drug resistance. To confirm this hypothesis, drug-induced apoptosis in chemotherapy was evaluated through transfecting SK-BR-3 and SNU-216 cells with miR-3622b-5p mimic. According to MTT assay, cells transfected with miR-3622b-5p mimic exhibited greatly decreased resistance to cisplatin and 5-fluorouracil (5-FU) compared with cells transfected with miRNA mimic control (Figure 7A and 7B). These results suggest that miR-3622b-5p weakens the resistance of ERBB2-positive cancer cells against cisplatin and 5-FU.

## DISCUSSION

MiR-21 signaling sustains epithelial-to-mesenchymal transition (EMT) in ERBB2-positive breast cancer [25]. MiR-125b is down-regulated in metastatic breast cancers and there is a positive correlation between ERBB2/HER2 level and erythropoietin receptor (as a target of miR-125b) level [26]. MiR-221 enhances the resistance against trastuzumab and the metastasis of ERBB2-positive breast cancer by targeting PTEN [27]. The expression of ERBB2 is inversely correlated with the level of miR-155 (a well-documented oncogenic miRNA) in ERBB2-positive breast tumors [19]. In this experiment, miR-3622b-5p was down-regulated in ERBB2-positive cancer tissues, suggesting its suppressive role in ERBB2-positive cancer.

When trastuzumab, small molecular targeted drug CP724714, or interfering RNA against ERBB2 are applied, the EMT-like phenotype of gastric cancer cells is dramatically reversed [28]. In breast cancer, the combination of 5-fluorouracil and cisplatin/docetaxel can trigger pathologic complete response, particularly in HER2-positive and triple-negative diseases [29]. The combination of irinotecan, 5-fluorouracil, leucovorin (FOLFIRI) and trastuzumab is curative for recurrent metastatic gastric cancer [30]. In this study, miR-3622b-5p made ERBB2-positive cancer cells more vulnerable to the apoptosis induced by cisplatin and 5-FU. Therefore, miR-3622b-5p can be taken as a novel target of chemotherapeutic agents.

In conclusion, miR-3622b-5p is involved in the proliferation and apoptosis of ERBB2-positive cancer cells via targeting ERBB2/mTORC1 signaling pathway. Therefore, miR-3622b-5p is a tumor suppressor in ERBB2-positive cancers and a potential diagnostic marker and therapeutic target for human ERBB2-positive cancers.

## MATERIALS AND METHODS

### Clinical samples

A total of 112 breast cancer patients and 71 gastric cancer patients recruited by the First Affiliated Hospital of Nanjing Medical University from 2013 to 2016 were included in this experiment. We collected 117 samples of cancer tissues and 30 of adjacent normal tissues, 71 of gastric cancer tissues and 30 of adjacent normal tissues. All tissues were histopathologically confirmed by a pathologist who selected areas of higher tumor cell density for RNA isolation and immunohistochemistry (IHC). All the samples were pathologically examined and stored in liquid nitrogen for miRNA analysis. Ethical approval for the study was granted by the Clinical Research Ethics Committee, Nanjing Medical University. Written informed consent was taken from each participant.

### Cell culture

SK-BR-3 human breast cancer cell lines, SNU-216 human gastric cancer cell lines, HEK-293T cell lines and MCF-10A normal human mammary epithelial cells were purchased from the Cell Bank of Shanghai (Shanghai, China). Cells were routinely cultured in RPMI 1640 medium or Dulbecco minimum essential medium (DMEM) medium, supplemented with 10% fetal bovine serum (Hyclone, Logan, UT, USA), at 37°C in a humidified atmosphere with 5% CO_2_.

### Quantitative real-time PCR analysis for miRNA

Breast cancer tissues and cells were isolated with Trizol reagent (Invitrogen, Carlsbad, CA) and miRNA fractions were further purified by mirVana™ miRNA isolation kit (Ambion, Austin, TX). The concentration and purity of RNA samples were determined spectroscopically. Expression of mature miRNA was assayed using stem-loop RT, followed by real-time PCR analysis. The SYBR and U6 genes were used for detecting the gene amplification and normalizing each sample, respectively. The primers of reverse transcription and polymerase chain reaction were purchased from RiboBio Co., Ltd (Guangzhou, China) named Bulge-Loop™ miRNA qRT-PCR Primer Set as previously described [31]. QRT-PCR was performed according to the protocol of primer sets. PCR amplification was detected by the level of fluorescence emitted by SYBR Green (SYBR^®^Premix Ex Taq™ II, TaKaRa) intercalated into double-stranded DNA [31]. The ΔCt method was used for miRNA expression analysis of biopsy specimens. First, the cycle number at the threshold level of fluorescence (Ct) in each sample was determined. Next, the ΔCt value was calculated to show the difference between Ct value of miR-3622b-5p and Ct value of U6: ΔCt = Ct(miR-3622b-5p) - Ct(U6). The fold-change for miR-3622b-5p was calculated using 2^−ΔΔCt^ method. PCR was performed in triplicate.

### RhoA and Rac1 activation assay

In RhoA and Rac1 activation assays (Cytoskeleton Inc., Denver, CO, USA), cells were seeded into 6-well plates and transfected with miR-3622b-5p mimic or miRNA mimic control. The experiments were then performed according to the manufacturer's protocol. RhoA and Rac1 activation assay was performed in triplicate.

### *In vitro* drug sensitivity assay

SK-BR-3 or SNU-216 cancer cells were seeded into 6-well plates (6×10^5^ cells/well), 100 nmol/L miR-3622b-5p mimic or 100 nmol/L miRNA mimic control was transfected by Lipofectamine 2000 (Invitrogen, Long Island, NY, USA) according to the manufacturer's protocol, respectively. The miR-3622b-5p mimic and miRNA mimic control were chemically synthesized by Shanghai GenePharma Company (Shanghai, China). Twenty four hours after transfection, cells were seeded into 96-well plates (5×10^3^ cells/well). Another forty eight hours after drug administration, cell viability was assessed by 3-(4,5-dimethylthiazol-2-yl)-2,5-diphenyl-tetrazolium bromide (MTT) assay. The absorbance at 490 nm in each well was read on a spectrophotometer. The concentration at which drugs produced 50% inhibition of growth (IC50) was estimated by the relative survival curve. Three independent experiments were performed in quadruplicate.

### Dual-luciferase activity assay

The 3′-UTR of human ERBB2 containing the putative target site of the miR-3622b-5p was synthesized and placed at the *Xba*I site in the pGL3-control vector (Promega, Madison, WI) by Integrated Biotech Solutions Co., Ltd (Shanghai, China). Twenty four hours before transfection, cells were seeded into 24-well plates (1.5×10^5^ cells/well). Then, 200 ng of pGL3-ERBB2-3′-UTR-WT or pGL3-ERBB2-3′-UTR-WT-del plus 80 ng pRL-TK (Promega) were transfected with 60 pmol miR-3622b-5p mimic or miRNA mimic control by using Lipofectamine 2000 (Invitrogen) according to the manufacturer's protocol. Luciferase activity was measured twenty four hours after transfection using Dual Luciferase Reporter Assay System (Promega). Firefly luciferase activity was normalized to renilla luciferase activity for each transfected well. Three independent experiments were performed.

### Western blot analysis

SK-BR-3or SNU-216 cancer cells were placed into 6-well plates (6×10^5^ cells /well). Seventy two hours after the transfection with miR-3622b-5p mimic or miRNA mimic control, cells were harvested and homogenized with lysis buffer. Total protein was separated by denatured 10% SDS-polyacrylamide gel electrophoresis. Western blot analysis was performed [32]. The primary antibodies for ERBB2, S6, Phospho-S6, â-actin and GAPDH were purchased from Cell Signaling Technology (Danvers, MA). The primary antibody for ERBB3 was purchased from Proteintech Wuhan Sanying (Wuhan, China). Protein levels were normalized to â-actin or GAPDH.

### Clonogenic assay

SK-BR-3 or SNU-216 cells were transfected with miR-3622b-5p mimic (with/without ERBB2 lacking 3`-UTR) and placed into 6-well plates (1000 cells/well), incubated at 37°C for 2 weeks, fixed and stained with crystal violet. The mean ±SD number of colonies was counted under a microscope from three independent replicates.

### Apoptosis assay

SK-BR-3 or SNU-216 cells were placed into 6-well plates (6×10^5^ cells /well). Forty eight hours after the transfection with miR-3622b-5p mimic (with/without ERBB2 lacking 3`-UTR), flow cytometry was used to detect the apoptosis of transfected cells by determining the amount of AnnexinV-positive and PI-negative cells.

### Statistical analysis

Each experiment was repeated for at least 3 times. Numerical data were presented as mean±SD. The difference between means was analyzed with Student's t-test. All statistical analyses were performed using SPSS 13.0 software (Chicago, IL). Differences were considered significant when *p*< 0.05.

## SUPPLEMENTARY MATERIALS FIGURES


