# Supplementary material for: Tumor suppressor role of miR-3622b-5p in ERBB2-positive cancer
Source: Oncotarget. 2017 Feb 1;8(14):23008–19. doi: 10.18632/oncotarget.14968 (PMC5410281; doi:10.18632/oncotarget.14968)
Supplement: Supplementary file 1 [file oncotarget-08-23008-s001.pdf]

# Tumor suppressor role of miR-3622b-5p in ERBB2-positive cancer

## SUPPLEMENTARY FIGURES

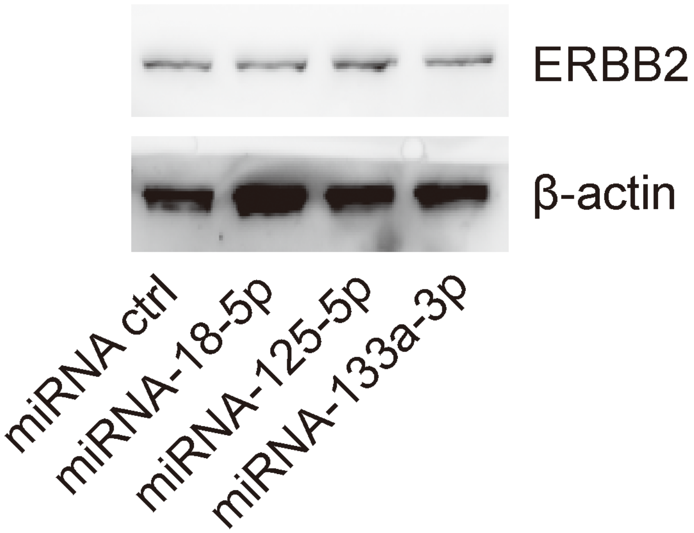


**Supplementary Figure 1: The overexpression of miR-18-5p, miR-125-5p or miR-133a-3p do not alter the expression level of ERBB2.** Western blot analysis showing unaltered protein levels of ERBB2 in SNU-216 gastric cancer cells after miR-18-5p, miR-125-5p or miR-133a-3p overexpression. β-actin as the internal control.

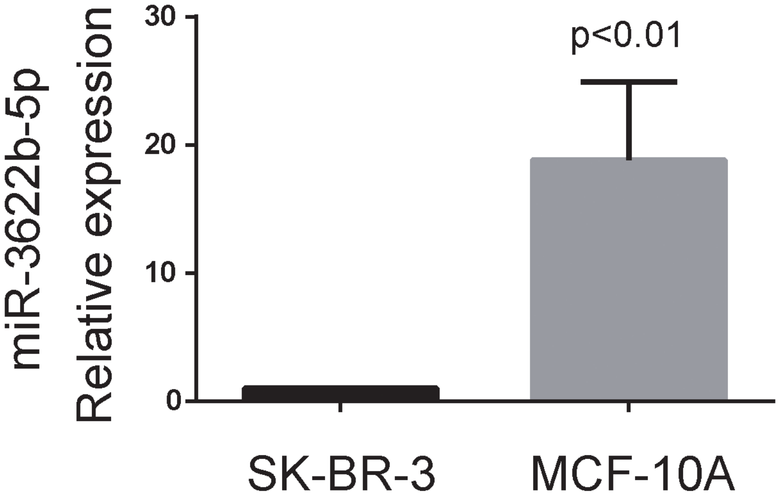


**Supplementary Figure 2: MiR-3622b-5p is down-regulated in ERBB2-positive breast cancer cells.** The miR-3622-5p expression is suppressed in SK-BR-3 ERBB2-positive breast cancer cells when compared to that in MCF-10A normal human mammary epithelial cells. The miRNA relative expression levels were normalized to the average value of SK-BR-3 breast cancer cells.
